# Neuroimmune activation is associated with neurological outcome in anoxic and traumatic coma

**DOI:** 10.1093/brain/awae045

**Published:** 2024-02-27

**Authors:** Benjamine Sarton, Clovis Tauber, Estéban Fridman, Patrice Péran, Beatrice Riu, Hélène Vinour, Adrian David, Thomas Geeraerts, Fanny Bounes, Vincent Minville, Clément Delmas, Anne-Sophie Salabert, Jean François Albucher, Benoit Bataille, Jean Marc Olivot, Alain Cariou, Lionel Naccache, Pierre Payoux, Nicholas Schiff, Stein Silva

**Affiliations:** Critical Care Unit, University Teaching Hospital of Purpan, F-31059 Toulouse Cedex 9, France; Toulouse NeuroImaging Center, Toulouse University, Inserm 1214, UPS, F-31300 Toulouse, France; Imaging and Brain laboratory, UMRS Inserm U930, Université de Tours, F-37000 Tours, France; Brain and Mind Research Institute, Weill Cornell Medical College, New York, NY 10065, USA; Toulouse NeuroImaging Center, Toulouse University, Inserm 1214, UPS, F-31300 Toulouse, France; Critical Care Unit, University Teaching Hospital of Purpan, F-31059 Toulouse Cedex 9, France; Critical Care Unit, University Teaching Hospital of Purpan, F-31059 Toulouse Cedex 9, France; Critical Care Unit, University Teaching Hospital of Purpan, F-31059 Toulouse Cedex 9, France; Neurocritical Care Unit, University Teaching Hospital of Purpan, F-31059 Toulouse Cedex 9, France; Critical Care Unit, University Teaching Hospital of Rangueil, F-31400 Toulouse Cedex 9, France; Critical Care Unit, University Teaching Hospital of Rangueil, F-31400 Toulouse Cedex 9, France; Cardiology Department, University Teaching Hospital of Purpan, F-31059 Toulouse Cedex 9, France; Toulouse NeuroImaging Center, Toulouse University, Inserm 1214, UPS, F-31300 Toulouse, France; Neurology Department, University Teaching Hospital of Purpan, F-31059 Toulouse Cedex 9, France; Critical Care Unit, Hôtel Dieu Hospital, F-11100 Narbonne, France; Neurology Department, University Teaching Hospital of Purpan, F-31059 Toulouse Cedex 9, France; Critical Care Unit, APHP, Cochin Hospital, F-75014 Paris, France; Institut du Cerveau et de la Moelle épinière, ICM, PICNIC Lab, F-75013 Paris, France; Toulouse NeuroImaging Center, Toulouse University, Inserm 1214, UPS, F-31300 Toulouse, France; Brain and Mind Research Institute, Weill Cornell Medical College, New York, NY 10065, USA; Critical Care Unit, University Teaching Hospital of Purpan, F-31059 Toulouse Cedex 9, France; Toulouse NeuroImaging Center, Toulouse University, Inserm 1214, UPS, F-31300 Toulouse, France

**Keywords:** disorders of consciousness, neuroimmune activation, TSPO PET scan, prognosis, traumatic brain injury, brain anoxia, mesocircuit

## Abstract

The pathophysiological underpinnings of critically disrupted brain connectomes resulting in coma are poorly understood. Inflammation is potentially an important but still undervalued factor. Here, we present a first-in-human prospective study using the 18-kDa translocator protein (TSPO) radioligand ^18^F-DPA714 for PET imaging to allow *in vivo* neuroimmune activation quantification in patients with coma (*n* = 17) following either anoxia or traumatic brain injuries in comparison with age- and sex-matched controls.

Our findings yielded novel evidence of an early inflammatory component predominantly located within key cortical and subcortical brain structures that are putatively implicated in consciousness emergence and maintenance after severe brain injury (i.e. mesocircuit and frontoparietal networks). We observed that traumatic and anoxic patients with coma have distinct neuroimmune activation profiles, both in terms of intensity and spatial distribution. Finally, we demonstrated that both the total amount and specific distribution of PET-measurable neuroinflammation within the brain mesocircuit were associated with the patient’s recovery potential.

We suggest that our results can be developed for use both as a new neuroprognostication tool and as a promising biometric to guide future clinical trials targeting glial activity very early after severe brain injury.

## Teaser

Sarton, Tauber *et al.* investigate the relationship between neuroinflammation, measured *in vivo* using TSPO PET, and neurological outcomes in patients with coma following cardiac arrest or traumatic brain injury.

See Tenovuo *et al*. (https://doi.org/10.1093/brain/awae082) for a scientific commentary on this article.

## Introduction

Coma, typically arising from traumatic or anoxic brain injury, is a major health issue worldwide; prolonged coma often predicts long-term, typically lifelong, cognitive and behavioural impairments.^1,2^ Despite recent developments in the characterization of structural and functional brain damage underlying coma, prediction of neurological recovery for these patients remains a conundrum, thereby necessitating a better understanding of brain injury mechanisms that are responsible for consciousness abolition in this setting^3-6^ and those supporting its recovery.^4^ Actually, filling this knowledge gap holds promise for both the discovery of accurate and actionable biomarkers of clinical outcomes and the development of highly needed new targeted patient-tailored interventions.

Regardless of aetiology, a growing body of evidence suggests that the common pathophysiological mechanism underlying coma is a broad withdrawal of excitatory synaptic activity across key neocortical and subcortical brain structures (i.e. mesocircuit and frontoparietal networks).^7,8^ However, the pathophysiological processes underpinning the disruption of these critical brain connectomes is poorly understood, but acute neuroinflammation might be one important factor. Indeed, it is well known that severe brain injury induces a significant central and systemic inflammatory response.^4,7,9,10^ Increasing evidence suggests that the neuroinflammatory counterpart of this adaptative mechanism could be located within brain regions that are the critical hubs of consciousness-related networks. Hence, a marked frontal, parietal and thalamic accumulation and activation of glial cells has been reported in post-mortem specimens from patients who died from either acute traumatic^11^ or anoxic^12^ brain injury, and animal models studies have identified early inflammatory processes in both traumatic^13^ and anoxic^14^ conditions, particularly within the associative cortices and sub-cortical grey matter.

At a cellular level, microglia are key players in the central immune response, in which they act as brain-resident macrophages.^15^ In response to brain insults, microglia are transformed from a sentry state into an active state and increase the expression of a mitochondrial protein, the 18-kDa translocator protein (TSPO). As a result, in response to microglial activation, TSPO is overexpressed, compared with its expression in normal tissues, making it a key marker of neuroimmune activation.^16^ In addition to their role in inflammation, microglia support neuronal viability and regeneration^17^ and play a critical role in brain plasticity after brain injury.^18^ However, it should be noted that chronic activation of microglia can become deleterious for neuronal cells and constitutes an important factor in neurodegenerative processes.^19^

Because of its ability to provide *in vivo* measurements of selected proteins at low concentrations, PET seems exceptionally well suited to *in vivo* quantification of neuroinflammation in brain injured patients. For example, TSPO radioligands for PET imaging are well-validated and widely used biomarkers of neuroinflammation to assess diverse CNS pathologies in preclinical and clinical studies.^20^ Although TSPO is predominantly expressed in the brain by microglia, expression by other cell types should be considered. TSPO was originally found in peripheral tissue but is also expressed in the brain by astrocytes and in the vascular endothelium.^20^ Migration of peripheral myeloid cells into the brain can also contribute to the TSPO signal.^21^ Furthermore, despite the fact that second-generation TSPO radioligands such as ^18^F-DPA-714 have improved the ratios of specific to non-specific binding, they are sensitive to a common polymorphism (rs6971) in the *TSPO* gene. Individuals with two copies of the rare allele (i.e. low-affinity binders) bind these radioligands with a lower affinity than people with two copies of the major allele (i.e. high-affinity binders) and people who are heterozygous for this allele (i.e. mixed-affinity binders) express both high-affinity and low-affinity binding sites in similar proportions. This obstacle has been addressed by performing *TSPO* genotyping, which allows the exclusion of low-affinity binders from further analyses. Despite these limitations, the study of PET-measurable neuroimmune activation has allowed the detection of significant neuroinflammation in a limited number of small *in vivo* brain imaging studies of individuals months to years after moderate to severe traumatic brain injury.^19,22,23^ Thereafter, a report of moderate to severe traumatic brain injury patients, who were scanned while awake at least 11 months after the primary brain insult, revealed that patients’ TSPO binding potentials were significantly higher than those of controls in the thalami, the putamen and the posterior limb of the internal capsule.^23^ However, to our knowledge, there is no *in vivo* evidence about neuroimmune activation in either anoxic or traumatic coma patients in the acute setting.

In this study, we sought to investigate the relationship between acute neuroinflammation detected *in vivo* and neurological outcome in patients with coma 90 days after primary brain injury. We hypothesized that brain injuries associated with coma trigger microglia within selective brain structures putatively responsible for both loss and recovery of consciousness (e.g. mesocircuit and fronto-parietal networks).^7,8^ We further anticipated that traumatic and anoxic patients with coma would demonstrate distinct *in vivo* microglial activation profiles, both in terms of intensity and spatial distribution, and that neuroimmune activation during coma would show a significant relationship with the patients’ further neurological outcome. Finally, in traumatic patients with coma, we also investigated PET-measurable neuroinflammatory signals within traumatic focal lesions, derived from structural MRI.

## Materials and methods

### Experimental design

We undertook a cross-sectional study of traumatic and anoxic patients with coma in comparison with age-matched controls. This prospective study was undertaken in three intensive care units affiliated with the University Hospital of Toulouse (Toulouse, France) between February 2018 and February 2022. TSPO PET (^18^F-DPA-714), MRI and clinical assessments (Glasgow Coma Scale, GCS; Full Outline of Unresponsiveness, FOUR)^24^ were performed at baseline. Patients were followed up at 3 months after primary brain injury, including scoring with the revised version of the Coma Recovery Scale (CRS-R).^7^ Our study was approved by the Ethics Committee of the University Teaching Hospital of Toulouse, France. Informed and written consent to participate to the study was obtained from the subjects themselves in the case of healthy subjects and from legal surrogates of the patients. Clinical trials identifier: NCT03482115.

### Population

**Figure 1:**
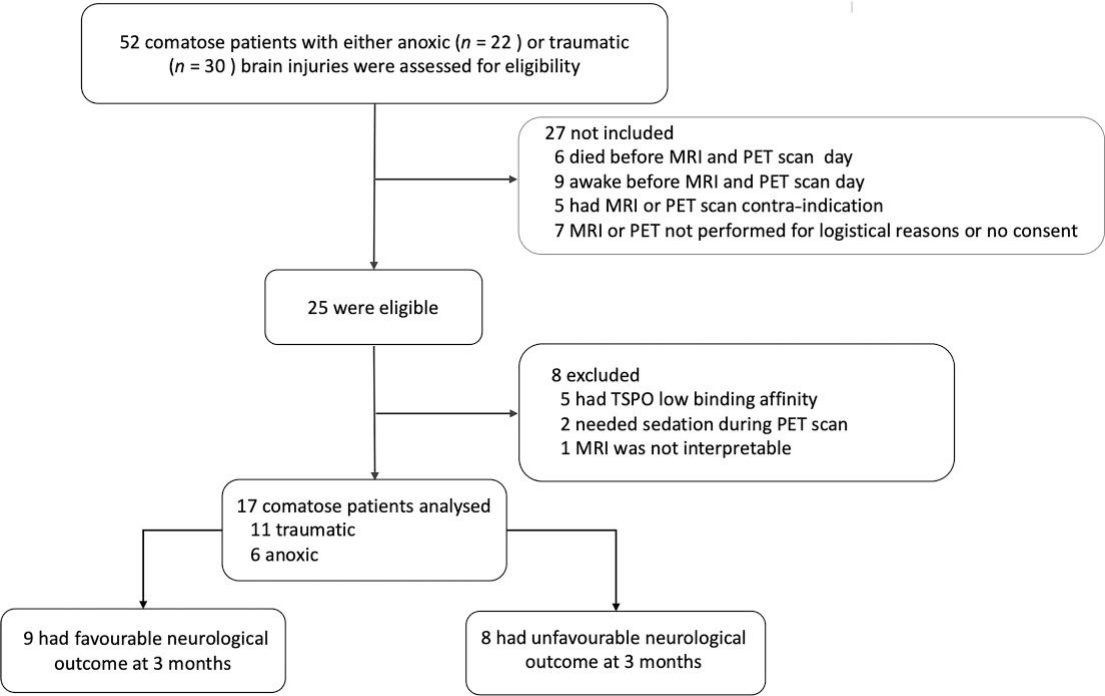
**Study flow chart**. TSPO: a third generation translocator protein (18 kDa) radioligand for PET imaging (^18^F-DPA714).

Patients were included in the study after a behavioural assessment using the GCS (GCS score at admission to hospital ≤9 with motor responses <6) and a diagnosis of coma induced by either severe traumatic or anoxic brain injury. Patients needed to maintain a GCS score ≤9 with motor responses <6 without sedation at the time of brain imaging. Patients not meeting this criterion at assessment were excluded (Fig. 1). Additional exclusion criteria were: significant neurological or psychiatric illness prior to coma; the use of steroidal or non-steroidal anti-inflammatory drugs before imaging; or a low affinity genotype for *TSPO* (rs6971 gene).^16,25^ Patients’ peripheral blood samples were drawn on the day of the TSPO PET scan to characterize their *TSPO* genotypes. Based on the rs6971 polymorphism within the *TSPO* gene,^26^ we classified all subjects into three groups: high-affinity binders; mixed-affinity binders; or low-affinity binders. TSPO PET scan data from low-affinity binder patients were discarded from further analysis^19,26-29^ (Fig. 1).

All patients were managed according to standard of care recommendations^30,31^ by physicians blinded to the neuroimaging data. To avoid confounding factors, all patient assessments were conducted at least 2 days (4 ± 2 days) after complete withdrawal of all sedative drug therapy and performed under normothermic conditions. On the day of brain imaging, urine benzodiazepine and barbiturate screening tests were used in patients to rule out residual sedation in cases of prolonged utilization of these drugs. Over the same recruitment period, 24 controls, matched by age and sex, were recruited. Control participants denied any history of substance dependence and current use of anti-inflammatory treatment, including over-the-counter medications. Participants were included if they had normal neurological examination results, no history of neurological or psychiatric disorders and had a *TSPO* (rs6971) genotype other than low-binding.

### Clinical assessment

In patients, standardized clinical examination (GCS and FOUR)^32,33^ was performed by raters blinded to the neuroimaging data on the day of patient’s admission to the hospital and the day of MRI and PET scanning.^34^ A patient’s neurological outcome was assessed 3 months after the primary brain injury using the CRS-R.^35^ The CRS-R is a 23-point scale measuring arousal level, auditory function, language, visuo-perception, communication abilities and motor function.^36^ This scale enables the distinction between patients in a vegetative state [VS, also coined unresponsive wakefulness syndrome (UWS)] and patients in a minimally conscious state (MCS) or patients who had emerged from a MCS (EMCS) and recovered consciousness as reflected by functional communication or functional use of objects. According to this scale, a patient’s 90 day outcome was binarized as either favourable (MCS or EMCS) or unfavourable (VS/UWS or deceased).

### MRI acquisition

For all participants, high-resolution anatomical images using 3D T1-weighted sequences were acquired on the same 3 T magnetic resonance scanner (Philips Achieva, Dstream). Monitoring of vital measures was performed by a senior intensivist throughout the experiment.

### PET image acquisition and preprocessing

PET scanning was performed using standard protocols at the Cyclotron Building, Toulouse University Hospital, Toulouse, France, using a hybrid PET/CT tomograph (Siemens, Biograph TruePoint 6.0). Thirty seconds after the start of emission scanning, a mean dose of 3.5 MBq/kg of ^18^F-DPA-714 (maximum dose: 377 MBq) was administered intravenously over 10 s into the antecubital vein. This was followed by a 60-min mode list emission PET scan acquired as 32 time frames.

Images were reconstructed into a total of 32 time frames, with the frame setting of six 10-s, eight 30-s, five 1-min, five 2-min and eight 5-min frames. Attenuation correction, time-of-flight information and point-spread-functions were incorporated in the PET reconstruction. The image volume was 336 × 336 × 148 with a pixel size of 1.018 mm and a slice thickness of 1.5 mm. All PET images were corrected for head movement with a frame-by-frame 3D image registration to a composite frame.

### PET image quantification

The quantification of PET images was performed with a simplified reference tissue model (SRTM2) approach, which avoids arterial blood sampling. Considering the widespread distribution of TSPO in the brain, the reference time activity curve was estimated with a supervised cluster analysis (SVCA), as no anatomical region can be *a priori* supposed to be devoid of specific binding. Based on a previously reported procedure,^26^ our pipeline for PET image quantification was composed of four main steps: (i) segmentation of the cortical and subcortical structures; (ii) creation of two sets of classes from controls participants for both high- and mid-affinity binders; (iii) linear unmixing of the PET voxels all subjects and creation of the reference curves; and (iv) reference-based quantification of individual PET images. Segmentation of the brain was carried out using the recon-all pipeline in Freesurfer on each subject’s T1-weighted MRI, which included the segmentation of the subcortical structures, the extraction of subcortical surfaces, spatial normalization onto the FreeSurfer surface template and parcellation of the cortical regions for two atlases: Destrieux and Desikan–Kiliany. The PET image of each subject was then registered to its own T1-weighted magnetic resonance (MR) image with rigid transformation using PMOD v4.2 (PMOD Technologies Ltd.). All the frames of the PET images were normalized by subtracting the mean activity of the brain in the frame and dividing by the standard deviation of the frame. The SVCA classes were created independently for the high- and mid-affinity binder subjects. Ten high-affinity binder control subjects and 10 mid-affinity binder control subjects were used for the creation of SVCA classes. Normalized curves of the typical blood, non-specific binding grey matter, white matter and specific binding grey matter were generated. The blood class was created using the 40 voxels showing the most activity within the first 3 min of acquisition. The grey and white matter class was derived from the supratentorial regions obtained from FreeSurfer previously eroded to limit the partial volume effect. Similarly the non-specific binding grey matter class was obtained from the cerebellum grey matter after erosion. Lastly, the specific binding grey matter class was obtained using the eroded thalamus region obtained from FreeSurfer. One high-affinity binder control participant and one mid-affinity binder control participant were excluded from the class creation as their typical kinetic curves presented a significantly different non-specific binding grey matter profile compared with the other controls. All normalized curves from the retained control subjects were averaged separately among the high- and mid-affinity binder participants to create the two sets of SVCA kinetic classes. Dynamic PET images of all controls, anoxic and traumatic subjects were normalized using each subject’s brain mask obtained from FreeSurfer and decomposed voxel-wise as weighted linear combinations of the kinetic classes using non-negative least squares. For each subject, four 3D weight maps were generated, corresponding to each of the four classes of their corresponding affinity genotype. A low-binding grey matter weight ratio was calculated and used to identify the voxels over 0.9 that were used to generate the *ad hoc* reference curve of each subject. Parametric non-displaceable binding potential (hereafter referred to as TSPO level) images were generated using SRTM2, where the efflux rate constant was first estimated and subsequently fixed globally. All methods were implemented and performed in MATLAB 2017b.

### Statistical 3D parametric mapping

All T1-weighted MR images were registered onto the Hammer’s template from PMOD v4.2 in Montreal Neurological Institute (MNI) space with non-rigid transformations. The estimated transformations were then applied to the TSPO parametric maps to register them in the same reference space. Brain differences in TSPO levels between the three groups (controls, anoxic coma, traumatic coma) were measured via a voxel-based analysis using unpaired Student’s two-tailed *t*-tests with *P*-values corrected for multiple comparisons using the Benjamini–Hochberg control of false discovery rate. 3D *z*-score maps with a threshold of *P* = 0.05 were generated along with *d*-value maps to evaluate the effect size (moderate, large or very large effect size for *d*-values lower than 0.80, between 0.80 and 1.20 or higher than 1.20, respectively). Hammer’s template from PMOD v4.2 software (PMOD Technologies Ltd.) was applied to the *z*-score and *d*-value maps to obtain cerebral *z*- and *d*-values.

Partial least squares regression (PLS-R) was used to investigate the association between a patient’s outcome and whole-brain and voxel-wise TSPO levels. Considering the very large number of brain voxels that each act as an independent factor, and the fact that many of these voxels can be redundant (collinear in homogeneous regions), classic multiple linear regression approaches are not suited to constructing a predictive model of coma CRS-R outcome. Conversely, PLS-R is well-suited to problems that involve a large set of collinear factors, and it does not require strict assumptions with regard to variable and residual distributions. In addition, PLS-R is suitable when the matrix of factors has more variables than observations. All the voxels of the brain were considered as input factors to estimate each patient’s outcome. To avoid overfitting, a few underlying latent factors (voxels) that accounted for most of the variation in the outcome were extracted. Converse to principal component analysis, the loading vectors and latent variables not only account for variations in the factors but also model the outcome as well as possible and remain directly interpretable. To enable better interpretation of the PLS-R models, the variable importance in projection (VIP) coefficients were calculated. These estimate the importance of each factor in the outcome prediction of the PLS model, with a VIP score >1 indicating an important contribution. As each factor corresponds to a voxel in the brain, the VIP values were reshaped as 3D VIP maps of the brain to visualize the regions where the TSPO levels contributed the most to the patient’s outcome classification model. Cross-validation was used to predict the CRS-R outcome of each patient, with a leave-one-out strategy.

### Volume of interest definition

To test our hypothesis, ^18^F-DPA-714 binding potentials were sampled from an ensemble of anatomically defined grey and white matter volumes of interest (VOIs), defined by the mesocircuit and frontoparietal network model^4^: brainstem, thalamus, caudate, globus pallidum, putamen, corpus callosum, anterior cingulate cortex (ACC), posterior cingulate cortex (PCC), mesial prefrontal cortex (mPFC) and precuneus. Because they have been reported as likely to be affected by anoxic brain injuries, the superior parietal lobule, cuneus and hippocampus were also considered as VOIs (Supplementary Fig. 1). In addition, bilateral precentral gyrus was used as the control brain region not typically related to conscious processing.^4,7^ VOIs containing focal brain injury were removed to conduct this analysis. However, in the four traumatic patients with focal brain injury, we investigated TSPO levels within the lesion, lesion penumbra, thalamus and normal ipsilateral and contralateral grey and white matter (Supplementary Fig. 2). We defined spheric sample VOIs with a 5 mm radius in and around the most prominent lesion (Supplementary Fig. 2). One VOI was placed centrally within the lesion and a second within tissue adjacent to the lesion that returned an abnormal T1 signal. Based on the severity of our cohort and previous reports of increased neuroimmune activation remote from focal damage in moderate to severe traumatic brain injury patients,^23^ spheric VOIs were placed in grey and white matter returning normal MR signals both in ipsilateral and contralateral brain hemispheres and thalami.

### Statistical analysis

In accordance with recent reports,^19,23,37^ we pooled the mixed affinity and high-affinity binders together and used the *TSPO* genotype as a covariate in all statistical analyses. Data were analysed using R, a software environment for statistical computing and graphics (http://www.R-project.org/). Normality distribution was tested using the Shapiro–Wilk test. Differences between groups were assessed using χ^2^ or Kruskall–Wallis tests, or two-way ANOVA, when appropriate. Mandatory normal assumptions for the use of parametric ANOVA were assessed and *post hoc* testing was performed with Tukey’s honestly significant difference (Tukey-HSD) test.

## Results

### Population

Between February 2018 and February 2022, 53 patients were enrolled. Among the excluded patients, five were low-affinity binders, two needed sedation during the PET scan and one was excluded due to failure of the MRI brain normalization process (Fig. 1). Therefore, we eventually compared 17 severely brain injured patients who met the clinical definition of coma (GCS score at hospital admission <9 with motor responses <6; 11 patients with traumatic and six with anoxic brain injuries; age range 18–65 and 21–79 years, respectively) with 24 age- and sex-matched controls (age range 19–69 years). The mean interval between coma onset and PET scan was 13 ± 8 days. Detailed demographic and clinical data are summarized in Table 1 and Supplementary Tables 1 and 2.

**Table 1:** Clinical and demographic data

Patient	Age, years	BI	GCS	TSPO	CRS-R	Standard MRI findings
P1	18	Anoxic	6 (E1V1M4)	HAB	UWS/3	Corpus callosum FLAIR hyperintensity
P2	65	Anoxic	9 (E4V1M4)	HAB	MCS/17	Striatal and cuneus FLAIR hyperintensity
P3	59	Anoxic	5 (E1V1M3)	HAB	UWS/5	Striatal, occipital cortex FLAIR hyperintensity
P4	59	Anoxic	3 (E1V1M1)	MAB	Death^a^	Striatal lesions
P5	30	Anoxic	4 (E1V1M2)	MAB	UWS/4	Striatal parieto-occipital FLAIR hyperintensity
P6	23	Anoxic	6 (E1V1M4)	MAB	EMCS/21	Normal
P7	39	TBI	4 (E1V1M2)	HAB	UWS/7	Thalamic petechiae; DAI; SAH
P8	23	TBI	7 (E1V1M5)	HAB	EMCS/23	Bi-hemispheric contusions; bi-frontal petechiae
P9	21	TBI	5 (E1V1M3)	HAB	EMCS/23	Corpus callosum contusion; DAI; SAH
P10	36	TBI	8 (E2V1M5)	HAB	EMCS/23	Bifrontal and corpus callosum contusion; SAH
P11	40	TBI	3 (E1V1M1)	HAB	EMCS/23	Frontal petechiae; SDH; SAH
P12	70	TBI	5 (E1V1M3)	HAB	MCS/17	Left temporal contusion; SDH; DAI; SAH
P13	79	TBI	8 (E3V1M4)	MAB	Death^b^	Frontal petechiae; DAI; SDH; SAH
P14	22	TBI	8 (E3V1M4)	MAB	EMCS/23	DAI; SAH
P15	32	TBI	8 (E3V1M4)	MAB	UWS/9	DAI; SAH
P16	69	TBI	6 (E1V1M4)	MAB	UWS/4	DAI
P17	21	TBI	7 (E2V1M4)	MAB	EMCS/23	DAI, SDH

Patients’ Coma Recovery Scale-Revised (CRS-R) subscales are available in the Supplementary material. BI = brain injury; DAI = diffuse axonal injury; FLAIR = fluid attenuated inversion recovery; GCS = Glasgow coma scale (E = eye; V = verbal; M = motor); MCS = minimally conscious state; SAH = subarachnoid haematoma from traumatic origin; SDH = subdural haematoma; TBI = traumatic brain injury; TSPO = 18-kDa translocator protein polymorphism encompassing high, mid and low radioligand affinity binders (HAB, MAB and LAB, respectively); UWS = unresponsive wakefulness syndrome.

^a^Patient P4: best neurological status before dying: coma (GCS = 3 with E1V1M1); cause of death: multiple organ failure.

^b^Patient P13: best neurological status before dying: UWS (CRS-R = 5); cause of death: septic shock.

### Neuroimmune activation profiles according to brain injury mechanisms

**Figure 2:**
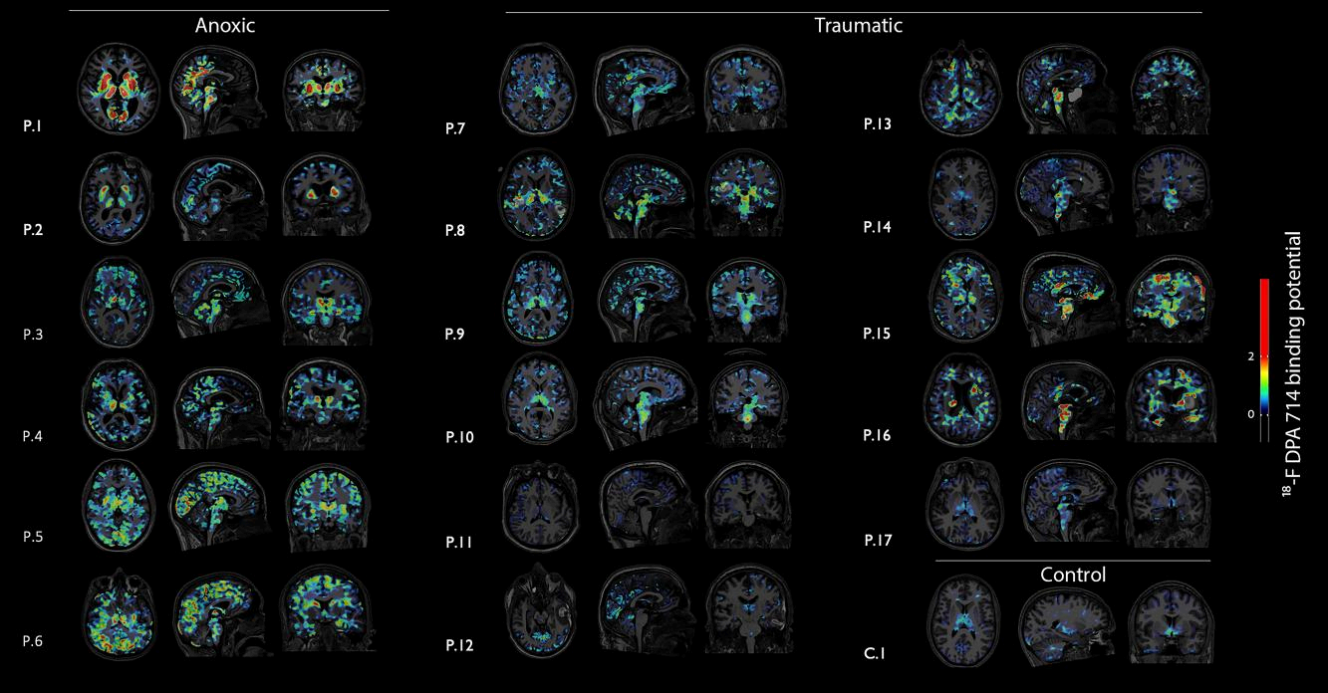
**Individual neuroimmune activation**. Translocator protein (18 kDa; TSPO) PET scan data depicted as co-registration with the subject’s native T1-weighted MRI. Six anoxic and 11 traumatic coma patients are shown. Brain images from one control subject are represented. Controls subjects and anoxic patient’s individual scans are depicted using canonical alignment to anterior and posterior commissure (ACPC). In a few cases, traumatic patients brain levels were displaced (±20 mm from ACPC) to better illustrate brain concussions and focal TSPO binding.

**Figure 3:**
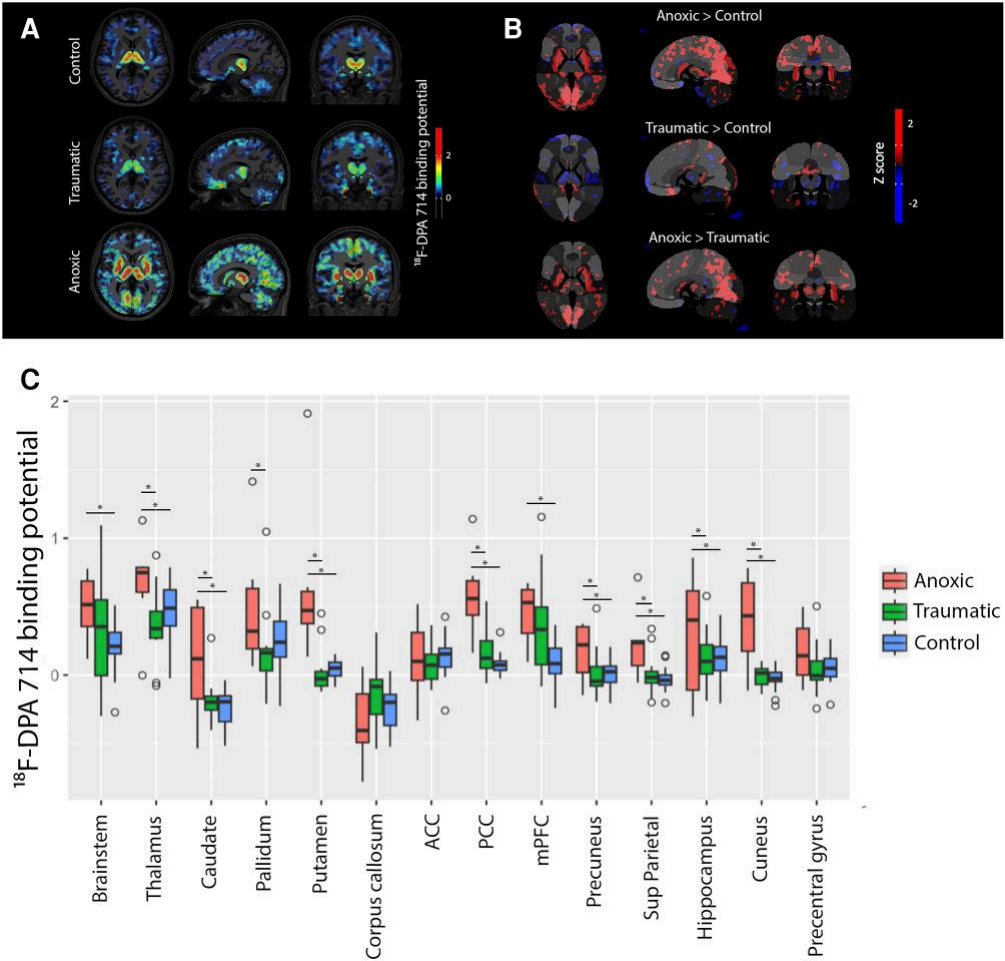
***In vivo* neuroimmune activation profiles according to primary brain injury mechanisms**. (**A**). ^18^F-DPA-714 binding potential, group analysis. (**B**) Whole-brain comparisons between comatose patient groups according to primary brain injury mechanism (*z*-scores). (**C**) Hypothesis-driven comparisons between comatose patient groups according to primary brain injury mechanism (volume of interest, VOI). *Corrected *P*-value < 0.05. ACC = anterior cingulate cortex; mPFC = medial prefrontal cortex; PCC = posterior cingulate cortex.

Whole-brain PET-measurable neuroinflammation was significantly greater in anoxic compared with traumatic patients with coma (Supplementary Fig. 3). Regions of abnormally high TSPO levels were found in most patients with coma compared with the controls (Fig. 2). However, the intensity and spatial distribution of these binding molecules significantly differed between the anoxic and traumatic patient groups. Indeed, a voxel-wise comparison of anoxic patients with coma versus controls showed a significant bilateral increase in ^18^F-DPA-714 binding in the thalamus, pallidum, putamen, PCC, mPFC, precuneus and cuneus (Fig. 3A and B). VOI analysis confirmed these observations (Fig. 3C). Indeed, we found a statistically-significant difference in average ^18^F-DPA-714 binding according to both coma aetiology [*F*(2) = 100.29, *P*-value < 10^−16^] and VOIs [*F*(12) = 14.03, *P*-value = 10^−16^], although the interaction between these factors was not significant. A Tukey-HSD test revealed significant pairwise differences between anoxic coma and control groups in all the studied VOIs: brainstem (*P*-value = 0.043); thalamus (*P*-value < 0.0001); caudate (*P*-value < 0.0001); globus pallidum (*P*-value < 0.0001); putamen (*P*-value < 0.0001); PPC (*P*-value < 0.0001); mPFC (*P*-value = 0.009); ACC (*P*-value = 0.043); precuneus (*P*-value = 0.005); cuneus (*P*-value < 0.0001); corpus callosum (*P*-value = 0.049); and hippocampus (*P*-value = 0.003). The *post hoc* pairwise comparison between traumatic patients with coma and controls only identified a significant TSPO levels difference in the mPFC (*P*-value = 0.02; Fig. 3). There was no significant difference in ^18^F-DPA-714 binding between the core and penumbra of traumatic focal lesions^23^ (Supplementary Fig. 2).

### Relationship between neuroimmune activation and neurological outcome

**Figure 4:**
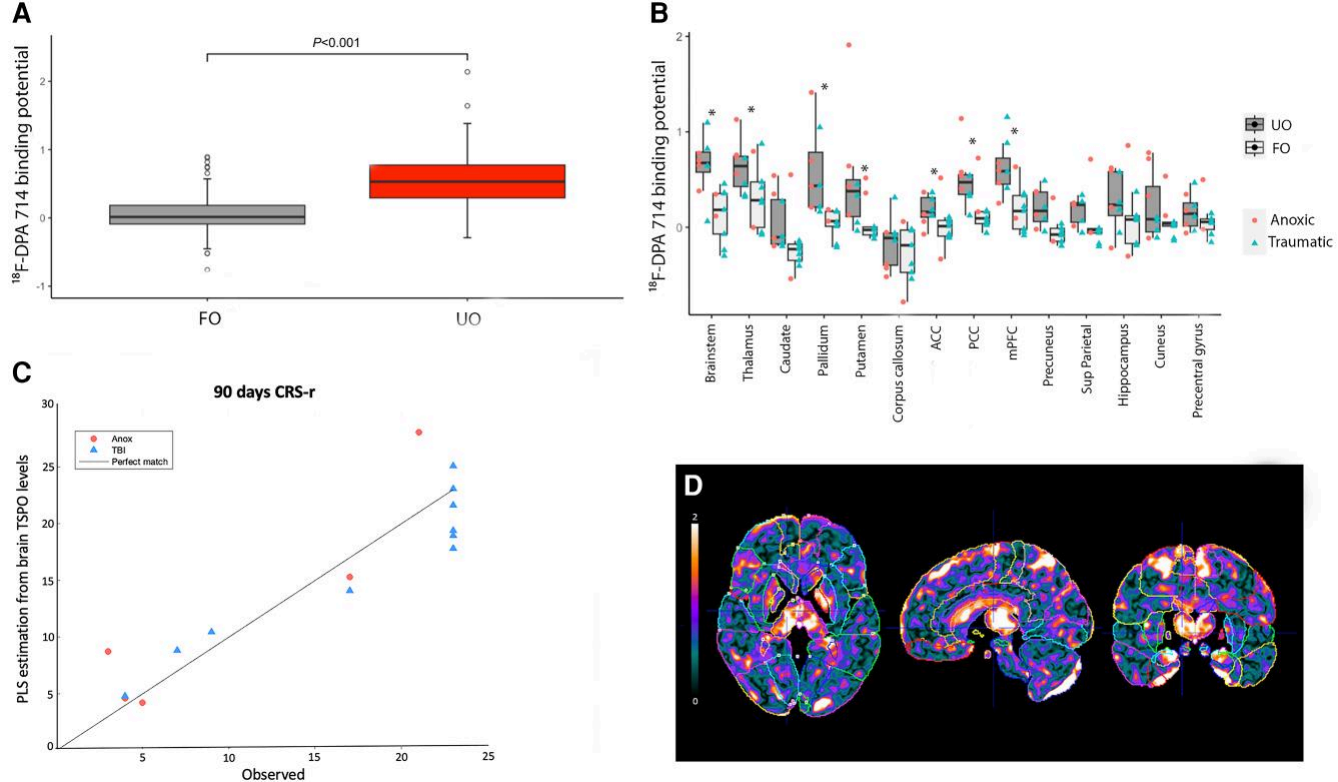
**Relationship between neuroimmune activation *in vivo* and coma patient’s neurological outcome**. (**A**). Whole-brain ^18^F-DPA-714 binding potential difference between patients with a favourable versus unfavourable neurological outcome at 3 months after coma onset. (**B**). Volume of interest ^18^F-DPA-714 binding potential difference between patients with a favourable versus unfavourable neurological outcome at 3 months after coma onset. (**C**) Estimation of patient Coma Recovery Scale-Revised (CRS-R) outcomes after 90 days using whole-brain voxel-wise cross-validated partial least squares (PLS) model [variable importance in projection (VIP) map]. The *x*-axis corresponds to observed outcomes and the *y*-axis to estimated outcomes. (**D**). Parametric map of VIP scores from the PLS regression. High values indicate an important contribution of the voxels to the model. ACC = anterior cingulate cortex; FO = favourable outcome; mPFC = medial prefrontal cortex; PCC = posterior cingulate cortex; UO = unfavourable outcome.

In patients, we investigated the relationship between baseline neuroinflammatory signals and subsequent neurological outcome at 6 months after primary brain injury. Significant higher TSPO levels were observed in coma patients with unfavourable outcome (Fig. 4A). Specifically, VOI analysis revealed significantly higher TSPO levels in patients with an unfavourable outcome compared with those that had a favourable outcome in the brainstem, thalamus, pallidum, putamen, caudate, PCC, mPFC, ACC and precuneus (Fig. 4B).

In addition, ANOVA allowed the identification of statistically significant differences in ^18^F-DPA-714 binding according to patient outcome [*F*(1) = 55.99, *P*-value < 10^−12^] and regions of interest [*F*(12) = 3.84, *P*-value = 10^−5^], however the interaction between these factors was not significant. *Post hoc* testing revealed significant pairwise differences between patient outcome groups at the level of the following VOIs: brainstem (*P*-value = 0.0032); thalamus (*P*-value = 0.032); globus pallidum (*P*-value < 0.0001); putamen (*P*-value = 0.007); PPC (*P*-value = 0.041); mPFC (*P*-value = 0.009); and ACC (*P*-value = 0.018). There was no interaction between patient outcome and coma aetiology. However, a significant correlation was observed between ^18^F-DPA-714 binding and patient behaviour as assessed by the CRS-R 3 months after primary brain injury in the pallidum (Spearman’s ρ = −0.73, *P*-value = 0.001), the putamen (Spearman’s ρ = −0.58, *P*-value = 0.02) and the PCC (Spearman’s ρ = −0.49, *P*-value = 0.04).

These findings were corroborated and expanded using a whole-brain and voxel-wise cross-validated predictive model (Fig. 4C). While in the model all the voxels are independently considered, the VIP maps (Fig. 4D) contained topological features with average value above 1 that can be interpreted as regions where the TSPO level best explains the patient outcome.

## Discussion

Recent research supports the idea that microglia, the resident immune cells of the CNS, play a major role in synaptic remodelling across the lifespan and after brain injury.^17^ Here, for the first time, we demonstrated *in vivo* that traumatic and anoxic brain injuries responsible for coma trigger specific neuroimmune activation across brain networks that are putatively implicated in conscious processing.^7,8^ Interestingly, we observed that distinct neuroimmune activation profiles were elicited by these two primary brain injury mechanisms. Last but not least, based on converging data from both VOI-based methods and robust data-driven whole-brain and voxel-wise statistical parametric mapping comparisons, we found a significant relationship between neuroimmune activation intensity and spatial distribution and patient neurological outcome. Overall, we consider that our results provide new insights into the characterization of neuroinflammation induced by severe brain injuries. Notwithstanding the observation that both the total amount and specific distribution of TSPO within patients’ brain mesocircuits were associated with the potential for neurological recovery, it is worth noting that our results do not provide any causal evidence between neuroimmune activation and patient outcome, and we acknowledge that these findings mostly reflect ongoing reparative processes^38^ and may simply be a reactive reflection of brain injury severity and not the driving force behind a patient’s further neurological status. Hence, our findings emphasize the need for future longitudinal studies tracking brain serial TSPO levels from coma onset in relation to neurological performance over time, aiming to understand whether these neuroimmune signatures persist, progress and/or warrant neuroimmune-modulating interventions.

To our knowledge, we have, for the first time, identified significant differences *in vivo* between the acute neuroimmune profiles induced by either traumatic of anoxic severe brain injuries. Notwithstanding that all patients were in coma at the time of the PET scan, each patient’s characteristics according to primary brain injury showed significant differences that might contribute to explaining the observed variations in TSPO levels at this group level. First, only clinically stable patients were enrolled, because particular attention was paid to patient safety during brain imaging. Consequently, the delays between coma onset and each patient’s neuroinflammatory state assessment were shorter and narrower in traumatic patients compared with cardiac arrest survivors. Second, due to the intrinsic diversity of primary brain injuries induced by severe brain trauma, the reported structural brain anomalies were more heterogenous both in terms of type and spatial distribution in the traumatic compared with the anoxic group. Third, our cross-sectional findings may reflect ongoing systemic inflammatory reactions to concomitant infections or extracranial traumatic injuries. Moreover, from a pathophysiological standpoint, we consider that in agreement with human post-mortem evidence,^12^ anoxic coma was associated with a significant expression of TSPO, which was mainly situated in brain regions belonging to the default mode network (DMN). Actually the DMN is known for being tonically active at rest,^39^ having the highest brain blood flow, oxygen consumption and oxygen extraction fraction at baseline,^40^ and therefore being particularly susceptible to energy delivery failure.^39^ Interestingly, in traumatic patients, we identified a different profile of neuroimmune activation, which was characterized by predominant innate immune cell activation within the frontal lobes, which are known for their vulnerability regarding traumatic brain injuries.^13,28^ It is worth noting that alternative mechanisms might also be responsible for the neuroimmune responses that we have observed. Secondary neurodegeneration,^27^ diaschisis,^41,42^ intracranial propagation of mechanical forces,^43^ and widespread endothelial cell activation and blood–brain barrier leakage might also contribute to both local and remote brain inflammation.^44^ Altogether, these observations and putative mechanisms underscore the need for specific characterization of TSPO expression in larger groups of patients with coma according to primary brain injury mechanisms.

Neurological impairment related to acute coma is now theorized as being contingent upon the structural^45-47^ or functional^48-50^ disruption of dynamic interaction between anterior forebrain mesocircuit and frontoparietal networks.^4,7^ Here we provided novel and converging evidence from both VOI-based and whole-brain voxel-wise analysis about the role of neuroimmune activation as a potentially major but still underrated mechanism of disturbance in key consciousness processing brain regions.^51^ Indeed, the association between patient outcome and their neuroinflammatory profiles that we have identified is consistent with the view that neuroinflammation can contribute significantly to the dysfunction of critical brain connectomes (i.e. inflammatory penumbra).^15,18^ Hence, it could be hypothesized that patient-tailored treatment approaches specifically designed to harness inflammation early-on after severe brain injury might reduce microglia-driven tau phosphorylation and neurofibrillary tangle formation,^15^ enhance repair and ultimately improve the neurological outcomes of patients with coma.

Our findings should be interpreted in light of the following potential limitations. First, we acknowledge that a patient’s concomitant sedation is a potential confounder for TSPO PET analysis^20,52^ and therefore might hinder the clinical applicability of this evaluation method in brain injured patients with disorders of consciousness. During the current study, we controlled this factor by scanning patients at least 2 days after total withdrawal of sedation and by testing urine benzodiazepine and barbiturate levels on the day of brain imaging, in case of previous utilization of these drugs. Moreover, two patients were later excluded because they required sedation during brain scanning. Second, despite the fact that both VOIs and whole-brain and voxel-wise analyses suggest a relationship between acute neuroimmune activation and unfavourable neurological outcome, we should keep in mind recent data which emphasize that TSPO expression in human myeloid cells is related to different phenomena than in mice, and that TSPO-PET signals in humans reflect the density of inflammatory cells rather than activation state^53^ and do not provide information about the dynamic proinflammatory/anti-inflammatory profile changes across time. Future studies should investigate, in larger cohorts of severe brain injured patients with and without disorders of consciousness, the beneficial or deleterious nature of the *in vivo* neuroinflammation that we have identified, probably by leveraging combined analyses of fluid-derived biomarkers and *in vivo* and *ex vivo* brain imaging. Another limitation is related to the global spatial distribution of neuroimmune activation and therefore the absence of a true reference region for inter-subject analysis. Here, we have circumvented the need for a kinetic model based on arterial sampling by using robust cluster sampling techniques.^26^ This approach has been successfully validated against arterial sampling methods and has been efficaciously used to identify microglial activation *in vivo* several years after moderate and mild traumatic brain injuries.^19,23,28^

Overall, our findings provide the first evidence that coma should be viewed as a condition with an early inflammatory component, which seems predominantly located in critical brain networks that are implicated in consciousness abolition and recovery after severe brain injuries. A significant spatial heterogeneity was observed between the neuroimmune activation profiles that were triggered by either traumatic or anoxic brain injury. We demonstrated that TSPO PET brain scans can be useful for exploring the association between *in vivo* neuroinflammation and severely brain injured patient outcome and think this approach can pave the way for the development of innovative personalized immunomodulatory therapeutics that ultimately permit us to breach the wall of decades of unconclusive and poorly stratified clinical trials in coma.^29,54^

## Supplementary Material

awae045_Supplementary_Data

## Data Availability

Anonymized data may be shared on request to the corresponding author from a qualified investigator for non-commercial use, subject to restrictions according to participant consent and data protection legislation.
