# Supplementary material for: Neuroimmune activation is associated with neurological outcome in anoxic and traumatic coma
Source: Brain. 2024 Feb 27;147(4):1321–30. doi: 10.1093/brain/awae045 (PMC10994537; doi:10.1093/brain/awae045)
Supplement: awae045_Supplementary_Data [file awae045_supplementary_data.pdf]

Supplementary Material

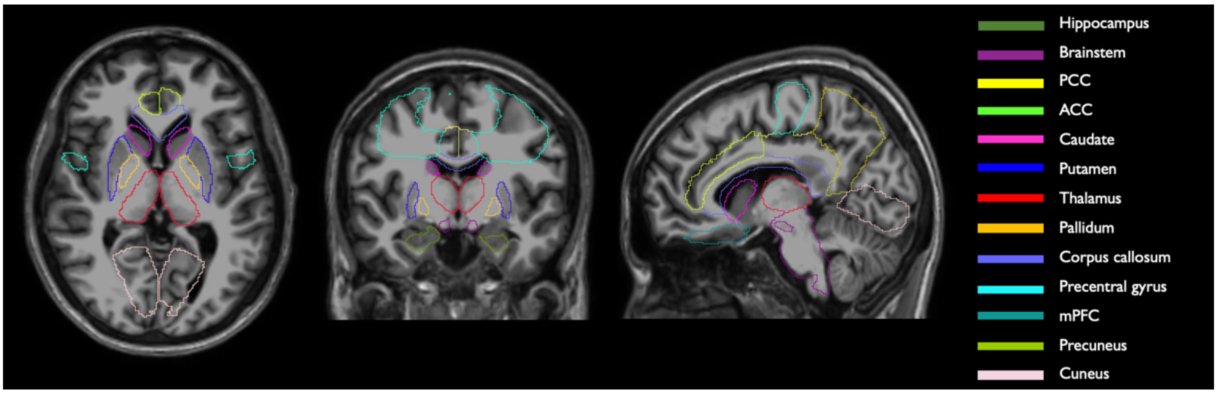

Fig.S1. Volumes of interest definition. Volumes Of Interest (VOI) from Hammers individual adult brain atlas[1,2]. Source www.brain-development.org. Abbreviations: PCC = posterior cingulate cortex; ACC = anterior cingulate cortex; mPFC = median prefrontal cortex.

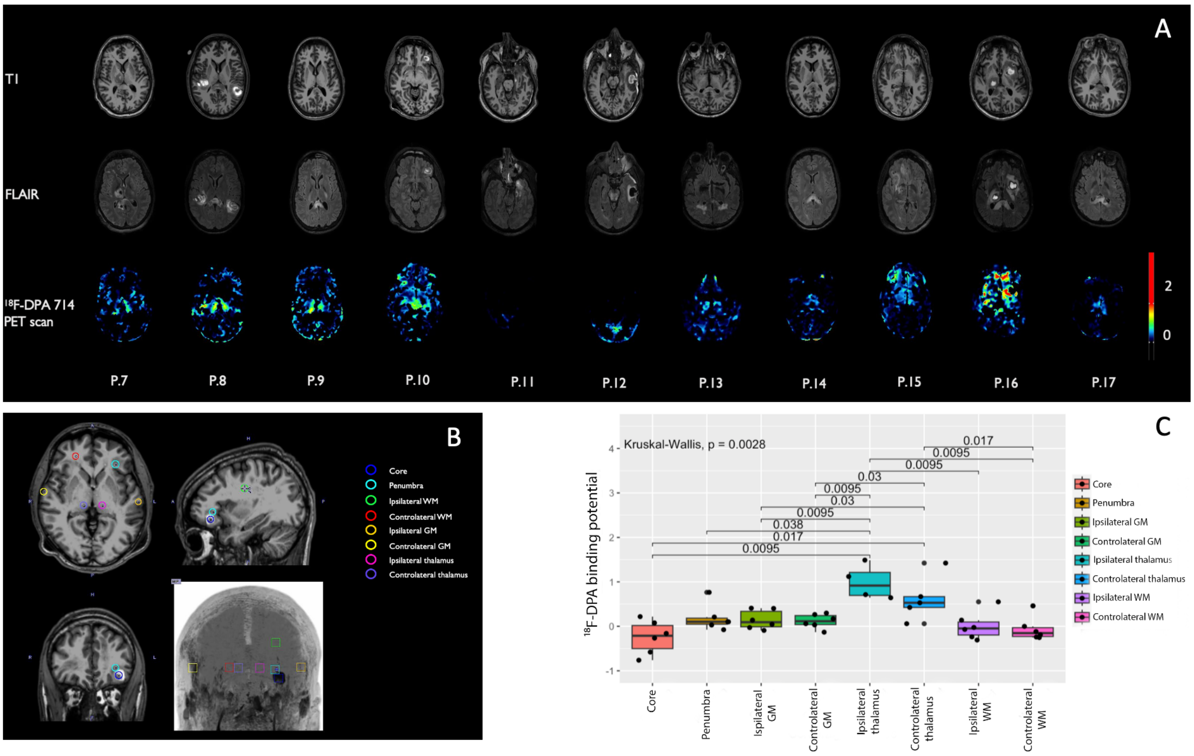

Fig.S2. **Focal lesion assessment**. A. T1, FLAIR and ^{18}F-DPA 714 PET scan data from the eleven traumatic brain injured patients with coma. B. Spheric volume of interest (VOI) of radius = 0.5 mm were used to probe TSPO levels within contusion core and penumbra and the ipsilateral and contralateral white matter (WM), grey matter (GM) and thalami. Illustration from patient 10. C. Mean values of 18F-DPA 714 PET within the pre-defined VOI.

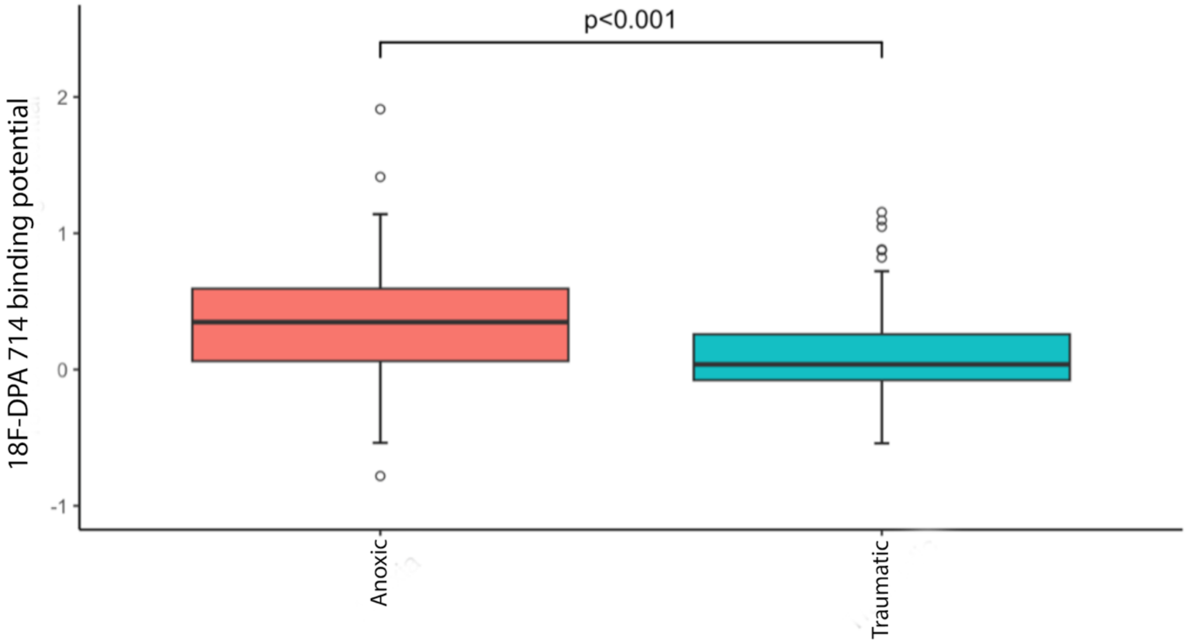

Fig.S3. Whole brain TSPO levels according to primary brain injury mechanism.

References

1. S. Gousias *et al.*, Automatic segmentation of brain MRIs of 2-year-olds into 83 regions of interest. *Neuroimage* **40**, 672-684 (2008).
2. A. Hammers *et al.*, Three-dimensional maximum probability atlas of the human brain, with particular reference to the temporal lobe. *Hum Brain Mapp* **19**, 224-247 (2003).

Anoxic patient's demographic and clinical data

Patient	Age	Sex	BI	Mechanism	Delay (BI to inclusion, days)	Medication (PET Day)	CRS-r subscale
P.1	18	F	Anoxic	CA / Drowning	30	Valproate, Ertapenem	A1 V0 M2 O0 C0 Ar0
P.2	65	F	Anoxic	CA / Hypoxia	30	None	A3 V4 M4 O2 C1 Ar3
P.3	59	M	Anoxic	CA / Epilepsy	8	Levetiracetam, Urapidil,	A1 V1 M1 O1 C0 Ar1
P.4	59	M	Anoxic	CA / Hypoxia	9	Furosemide, SA,	NA
P.5	30	F	Anoxic	CA / Cardiac ischemia	8	Levetiracetam, Valproate	A1 V0 M2 O0 C0 Ar1
P.6	23	M	Anoxic	CA / Commotio cordis	10	AC	A4 V5 M6 O2 C1 Ar3

Tab S1. The CRS-R encompass six subscales: A= Auditory;V= Visual; M. Motor; O= Oromotor; C= Communication. Ar=Arousal Functions. Abbreviations: AC= amoxicillin-clavulanate; BI= Brain injury; CA= Cardiac arrest; SA= salicylic acid.

Traumatic patient's demographic and clinical data

Patient	Age	Sex	BI	Mechanism	Extracranial traumatic injury	Delay (BI to inclusion,	Medication (PET Day)	CRS-r subscale
P.7	39	F	TBI	NP; MVRI	None	4	SA, Morphine	A1 V1 M2 O1 C0 Ar2
P.8	23	M	TBI	NP; MVRI	Femoral fracture	11	Meropenem, Linezolid	A4 V5 M6 O3 C2 Ar3
P.9	21	F	TBI	NP; Blunt /	Blunt chest trauma (hemothorax)	16	Cefepime	A4 V5 M6 O3 C2 Ar3
P.10	36	M	TBI	NP; MVRI	Orthopedic fractures	10	TZP, acid acetylsalicylic	A4 V5 M6 O3 C2 Ar3
P.11	40	M	TBI	NP; MVRI	Blunt chest trauma (ribs fractures)	11	AC	A4 V5 M6 O3 C2 Ar3
P.12	70	M	TBI	NP; Blunt /	Blunt chest trauma (ribs fractures)	6	AC, Urapidil	A3 V3 M4 02 C2 Ar3
P.13	79	M	TBI	NP; MVRI	Complex pelvic and orthopedic fractures	20	SA,	NA
P.14	22	F	TBI	MVRI	Liver and splenic trauma	9	Ceftriaxone	A4 V5 M6 03 C2 Ar3
P.15	32	M	TBI	MVRI	Mesenteric trauma	17	Propranolol	A2 V2 M2 O1 C0 Ar2
P.16	69	M	TBI	MVRI	Femoral fracture	16	Daptomycin,	A1 V0 M2 O1 C0 Ar0
P.17	21	M	TBI	MVRI	Blunt chest trauma (hemothorax)	5	TZP, propranolol	A4 V5 M6 O3 C2 Ar3

Tab S2. The CRS-R consists of 6 subscales: A= Auditory;V= Visual; M. Motor; O= Oromotor; C= Communication. Ar=Arousal Functions Abbreviations: AC= amoxicillin-clavulanate; BI= Brain Injury ; NP= Non penetrating; MVRI= Motor related vehicle injury ; SA= salicylic acid; TZP: Piperacillin-tazobactam.
